# Prospective Study on the Incidence and Progression of Clinical Signs in Naïve Dogs Naturally Infected by *Leishmania infantum*

**DOI:** 10.1371/journal.pntd.0002225

**Published:** 2013-05-09

**Authors:** Valentina Foglia Manzillo, Trentina Di Muccio, Sivia Cappiello, Aldo Scalone, Rosa Paparcone, Eleonora Fiorentino, Manuela Gizzarelli, Marina Gramiccia, Luigi Gradoni, Gaetano Oliva

**Affiliations:** 1 Department of Veterinary Clinical Sciences, University Federico II, Naples, Italy; 2 Unit of Vector-borne Diseases and International Health, MIPI Department, Istituto Superiore di Sanità, Rome, Italy; Ege University, Turkey

## Abstract

The incidence of clinical and clinicopathological signs associated with the progression of infection was evaluated prospectively in 329 naïve young dogs exposed to *Leishmania infantum* transmission and examined periodically during 22 months (M). The dogs were part of *Leishmania* vaccine investigations performed under natural conditions. Vaccinated groups were considered in the evaluation when the vaccine resulted non-protective and the appearance and progression of signs did not differ statistically from controls at each time point, otherwise only control groups were included. 115 beagles were part of 3 studies (A to C) performed in the same kennel; 214 owned dogs (29 breeds, 2.3% beagles) were included in a study (D) performed in 45 endemic sites. At M22 the prevalence of any *Leishmania* infection stage classified as subpatent, active asymptomatic, or symptomatic was 59.8% in studies A–C and 29.2% in study D. Despite different breed composition and infection incidence, the relative proportion of active infections and the progression and type of clinical and clinicopathological signs have been similar in both study sets. All asymptomatic active infections recorded have invariably progressed to full-blown disease, resulting in 56 sick dogs at M22. In these dogs, lymph nodes enlargement and weight loss — recorded from M12 — were the most common signs. Cutaneous signs were seen late (M18) and less frequently. Ocular signs appeared even later, being sporadically recorded at M22. Most clinicopathological alterations became evident from M12, although a few cases of thrombocytopenia or mild non-regenerative anemia were already observed at M6. Albumin/globulin inversions were recorded from M12 and urea/creatinine increase appeared mostly from M18. Altogether our findings indicate that any susceptible young dogs naturally infected by *L. infantum* present a common pattern of progression of signs during 2 years post infection, providing clues for medical and epidemiological applied aspects.

## Author Summary

Despite dogs representing the main reservoir for the most widespread entity of human leishmaniasis (zoonotic visceral leishmaniasis caused by *Leishmania infantum*, endemic in five continents), many aspects of canine leishmaniasis (CanL) are still poorly understood. Dog responses to the infection are complex and broadly follow the two patterns of substantial resistance or clinical susceptibility. The latter is a progressive condition eventually leading to a dog's death, being associated with an increasing infectiousness to phlebotomine vectors. Because most information on CanL comes from cross-sectional investigations such as in-clinic pet diagnosis or mass population surveys, knowledge on the individual natural course of the infection/disease is limited. Prospective investigations are rarely performed because they are difficult and expensive. We took advantage of 4 prospective studies on experimental *Leishmania* vaccines to investigate the time-course of clinical signs in susceptible young dogs exposed to natural infection. We found that, whatever the dog breed and the local incidence of leishmaniasis, any asymptomatic active infections detected in naïve animals invariably progress to full-blown disease, showing a common pattern of early and late signs during 2 years post infection. These findings may provide clues for medical and epidemiological applied aspects.

## Introduction

Zoonotic visceral leishmaniasis is a sand fly-borne disease caused by the protozoan parasite *Leishmania infantum* and is widely distributed in temperate and subtropical countries of both the Old and New Worlds [1]. In most of this range domestic dogs are the main reservoir host for human disease. Dogs may suffer from a severe disease (canine leishmaniasis, CanL) characterized by chronic evolution of viscero-cutaneous signs, such as lymph node enlargement, skin lesions, splenomegaly, onychogryphosis, renal and ocular damage due to immune complex deposition [2]. In endemic settings, a susceptible fraction of infected dogs - usually less than 50% - tends to progress towards clinical disease, whereas a resistant fraction does not [3]. However both seropositive asymptomatic dogs and dogs exhibiting clinical signs (“symptomatic”) can be infectious to phlebotomine vectors and contribute to a different extent to parasite transmission [4]–[6]. Once established in susceptible dogs, *L. infantum* infections progress over a variable period of time towards clinical conditions that can be highly diverse. Hence, attribution of CanL cases to specific clinical stages is not an easy task. Diagnosis of leishmaniasis in dogs should be based on an integrated approach including signalment, history, external clinical findings, and findings from basic laboratory analyses that may reveal direct or indirect damage by the parasite, or evaluate the immune responses of the host [7]. Nevertheless, once leishmaniasis is diagnosed veterinarians should be able to ascribe the infection or disease to a parasitological and clinical stage in order to establish adequate treatments or to predict progression toward more serious and irreversible stages [8].

To investigate how the appearance of clinical and clinicopathological signs over the course of time are associated with the progression of infection, we have carried out a retrospective analysis of the findings from a series of prospective CanL investigations sharing unique design features, such as i) the initial naïve condition of the study dogs, ii) a natural exposure to *Leishmania* infection at the age of 4–8 months, iii) a follow-up period long enough to include two consecutive sand fly transmission seasons, and iv) the performance of periodical parasitological, serological and clinical assessments carried out by the same laboratories and using the same methodologies.

## Materials and Methods

### Ethics statement

The study design and technical protocol of investigations on canine *Leishmania* vaccines were approved by the Veterinary Board of the Italian Ministry of Health following the European Directive 86/609/EEC, adopted by the Italian Government with the Law 116/1992.

### Study design

The analysis was performed on parasitological and clinical findings from 4 studies (here named as A, B, C and D) which were carried out to evaluate the efficacy of experimental *Leishmania* vaccines under natural transmission conditions. Vaccine efficacy results from study A have been published in full [9], [10]; those from study B have been communicated to an international veterinary meeting [11]; vaccine results from C and D studies have remained unpublished. Except for study D, the other studies consisted of blinded investigations, so that a retrospective analysis was only possible at the study termination when group allocation was disclosed. For those investigations (A, C and D) in which experimental vaccination did not confer any obvious protection as regards infection parameters and appearance/progression of clinical signs (see Results), both treated and untreated groups have been considered in the present analysis. For study B, which on the contrary demonstrated a significant vaccine-related clinical protection [11], only untreated control dogs have been taken into consideration. In fact, by the end of this study 6/20 control dogs developed symptomatic disease in contrast with 1/23 vaccinated dogs (p<0.05). As per protocols approved by the Italian Ministry of health and local authorities, the study dogs did not receive anti-*Leishmania* drug treatments if they showed clinical manifestation of CanL infection during the study.

### Places and dogs

Studies A, B and C were performed on Beagles housed in the same open-air kennel sited in a rural municipality of the Naples province (southern Italy) where both human visceral leishmaniasis and CanL are highly endemic. *Leishmania* seroprevalence in the local canine population was reported to be around 40% and the phlebotomine vector, *Phlebotomus perniciosus*, was found active from the end of May through the end of October [12]. Altogether 115 dogs were investigated, consisting of 7–8-month old animals born in non-endemic places of north Europe, and confirmed to be CanL-free through *Leishmania* serology (IFAT) and bone-marrow PCR examination at the time they were moved to the study site. In particular, study A included 45 dogs (22 males; 30 vaccinated and 15 controls), study B 20 dogs (11 males; controls only), and study C 50 dogs (25 males; 25 vaccinated and 25 controls). The animals were kept under constant veterinary care during the study period. The use of topical or environmental insecticides was avoided to allow natural exposure of dogs to sand fly bites in the warm season, however environmental and mechanical control of ticks and flea infestations was routinely performed. The dogs had received routine vaccination against leptospirosis, distemper, adenovirus, hepatitis, parainfluenza and parvovirus and were submitted to routine deworming treatment every six months.

Study D was performed in 45 sites of two contiguous Regions of southern Italy, namely Campania (42 sites) and Apulia (3 sites). Although these Regions are known to be endemic for CanL, accurate *Leishmania* seroprevalence rate in the general canine population of investigated sites was not available. The study included an initial number of 214 owned dogs (118 vaccinated, 106 controls) of different sex and breeds, aging 4–7 months at the beginning of the investigation. They were born from clinically healthy bitches during the winter season (December–March) preceding the study. Because in southern Italy phlebotomine vectors are not active during winter, and maternal *Leishmania* transmission by asymptomatic bitches has never been reported, we assume that these dogs were initially naïve. They belonged to 29 breeds (38% mongrel dogs) and consisted of pets, hunting or guard dogs living in different environmental conditions. At the inclusion, the dogs had a history of full routine vaccination and deworming; they were also confirmed *Leishmania* IFAT-negative. The use of commercial insecticide products labeled for the prevention of sand fly bites [13] was forbidden in case of treatments against fleas and ticks. *Leishmania* infections analysed in this paper developed in dogs from 12 sites, of which 10 in Campania and 2 in Apulia Regions.

### Study periods and assessment schedule

Study A was performed between June 2002 and April 2004; study B between June 2007 and April 2009; study C between July 2009 and April 2011; and study D between June 2006 and June 2008. Hence, all 4 studies included two consecutive transmission seasons, and were terminated just before or at the beginning of the third sand-fly season.

In the present analysis, studies A, B and C were grouped as a single “large” study because performed with dogs of same breed and age, and living in the same site whose environmental conditions determined quite a constant incidence of *Leishmania* infections during 9 years of investigations. On the other hand, study D could be considered as a comparative/confirmatory study, because involving dogs of different breeds (although of similar young age) and living in different environments.

In order to unify the gathered data and allow legitimate comparisons between different assessments, only the following time points have been considered in this analysis: month (M) 0, corresponding to the enrollment of dogs shortly before the first transmission season; M6, M12, M18 and M22 corresponding to complete serological, parasitological and clinical evaluations performed from initial sand fly exposure. At each time point the following biological samples were obtained: peripheral blood for IFAT serology, full blood count, total proteins, albumin/globulin ratio, and urea and creatinine value determination; sternal bone marrow (BM) aspirate for *Leishmania* DNA detection by nested polymerase chain reaction (n-PCR); aspirate from both popliteal lymph nodes (LN) for parasite culture. Before samplings, a clinical assessment was performed by accurate inspection of dogs for the presence of any external signs attributable to *Leishmania* infection. The collection of biological samples was performed in accordance with the national guidelines for animal welfare and, for study D, only after owner's informed consent.

### Parasitological and serological assays

Detection of anti-*Leishmania* IgG antibodies was performed by an in-house IFAT assay using *L. infantum* promastigotes (WHO reference strain MHOM/TN/1980/IPT-1) as antigen and following the protocol recommended by the Office International des Epizooties [14]. The cut-off dilution was set at 1∶160. BM aspirate material was examined by nested-PCR assay, as previously described [9]. Briefly, BM DNA was subjected to two consecutive PCR amplifications using the kinetoplastid-specific primers R221 and R332 in the first run, and the *Leishmania*-specific primers R223 and R333 in the second run [15]. LN aspirate material was cultured in parallel from each popliteal node in Evans' Modified Tobie's medium and cultures were periodically examined for promastigote growth during one month.

### Clinical evaluation

Any recorded clinical sign attributable to *Leishmania* infection was assigned to each of the following five categories:

- Systemic signs (S), including weight loss, muscular atrophy, lethargy and pale mucous membranes;
- Reticulo-endothelial signs (RE), including enlargement of palpable lymph nodes such as mandibular, prescapular or popliteal nodes, and spleen enlargement as determined by palpation;
- Cutaneous signs (C), including nodules/ulcers, onychogryphosis, dry exfoliative dermatitis and alopecia;
- Ocular signs (O), including blepharitis, keratoconjunctivitis and uveitis;
- Clinicopathological signs (CP), including anemia (hematocrit value <37%), thrombocytopenia (platelets count <200×10^3^/µL), increase of total proteins (>7,7 g/dL), hyperglobulinemia (albumin/globulin ratio <0.6), and increase of urea (>50 mg/dL) and creatinine (>1.5 mg/dL).

By the comparative analysis of clinical, serological and parasitological findings, the dogs were either considered non-infected (negative) or infected; any *Leishmania* infection detected at each assessment was assigned to one of the following stages (modified from [10]):

- Subpatent infection: detection of parasite DNA in BM samples; IFAT titers <1∶160; negative LN culture; absence of clinical and clinical-pathological signs attributable to CanL;
- Asymptomatic active infection: detection of parasite DNA in BM samples; IFAT titers ≥1∶160; positive LN culture; absence of clinical and/or clinical-pathological signs attributable to CanL;
- Symptomatic active infection: detection of parasite DNA in BM samples, IFAT titers >1∶160; positive LN culture; presence of clinical and/or clinical-pathological signs attributable to CanL.

The acute onset of possible arthropod-borne co-infections (such as ehrlichiosis, anaplasmosis, or babesiosis) was monitored by frequent recording of relevant clinical signs such as fever, depression, and respiratory distress. When a chronic condition of *Ehrlichia canis* infection was suspected (e.g. in case of severe thrombocytopenia) a specific IFAT test was performed [16].

### Statistical analysis

Data obtained at each time point were considered and the resulting proportions and frequencies of infection stages or clinical categories were compared for significance using the Chi square test, Fisher's exact test or Mann-Whitney test where appropriate, at a significance level of p≤0.05.

## Results

### Combined A, B and C studies

**Figure 1:**
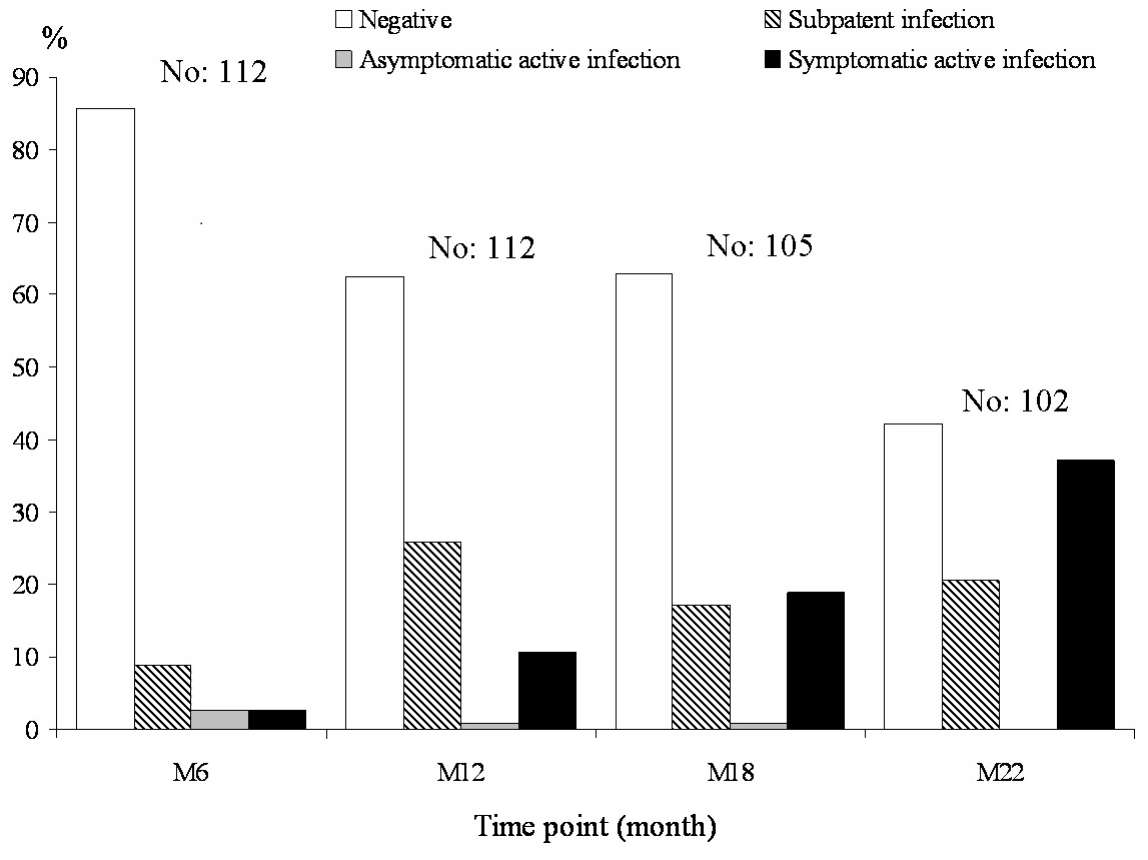
Combined prospective studies A, B and C. Incidence of *Leishmania* infection stages detected in 112 Beagle dogs at four time points from natural exposure.

A total of 102/115 Beagles completed the studies; 13 dogs died during the follow-up: 8 for causes unrelated to *Leishmania*, and 5 for severe CanL that became manifest from M6 in some dogs and led to the animal's death between M12 and M18 (3 dogs) or between M18 and M22 (2 dogs). Infection stages recorded at each time point are shown in Fig. 1. By M22, 43 dogs were still negative, 21 were in a subpatent infection stage, and 38 showed symptomatic active infection. By including the 5 dogs dead from CanL, the cumulative incidence of clinical disease was 43 out of 107 evaluable dogs (40.2%) in about 2 years from initial exposure. Subpatent infections were detected at each assessment and showed markedly different evolution patterns in subsequent assessments, including intermittent or steady subpatent condition, definitive conversion to negative or progression to active infection (data not shown). All asymptomatic active infections recorded during the studies, including those from time points not considered in this analysis, have invariably progressed to full-blown disease by the end of the observations.

**Figure 2:**
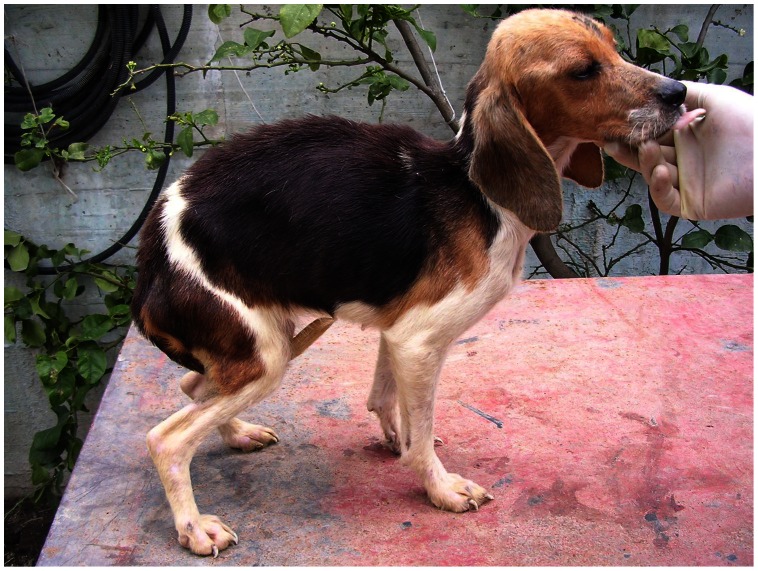
Leishmaniasis in a Beagle dog. Severe weight loss and muscular atrophy caused by active infection with *Leishmania infantum* was recorded at month 18 from natural exposure.

For the 43 dogs that developed symptomatic active infection, individual clinical and clinicopathological signs were recorded at the time of their appearance and categorized as reported in Methods (Table 1). As expected, the frequency and variety of clinical signs increased during time. The most frequent sign was the enlargement of lymph nodes that appeared at M12 and increased in frequency till involvement of 20/38 sick dogs survived at M22. Weight loss was the most frequent systemic sign involving up to 50% of sick dogs recorded at M18 (Fig. 2). Cutaneous signs, that included all the considered features, were typically late (from M18) and much less frequent, being recorded in only 8 sick dogs by the end of the study. Ocular signs were even rarer and appeared very late (one dog, recorded at M22). The large majority of clinicopathological alterations appeared after one year of observation. The earliest signs (M6, 4 dogs) were thrombocytopenia and/or mild non-regenerative anemia. Both signs increased progressively in frequency and eventually involved 87% and 71% of sick dogs survived at M22, respectively. Inversion of albumin/globulin ratio was recorded later (M12), but showed a similar trend; this sign was not always associated with an increase of total proteins. An increase of urea or creatinine, not always associated, appeared later and were the least frequent signs detected in survived sick dogs (29% and 11%, respectively).

**Table 1:** Incidence of clinical and clinicopathological signs, combined prospective studies A, B and C.

Sign category	Individual sign	No. of dogs showing signs and no. of signs recorded at each time point (M)			
		No = 3 (M6)	No = 12^a^ (M12)	No = 20^b^ (M18)	No = 38 (M22)
S	Weight loss	0	1	10	11
	Muscular atrophy	0	0	5	3
	Lethargy	0	0	0	0
	Pale mucous membranes	1	1	1	4
RE	Lymph node enlargement	0	5	10	20
	Splenomegaly	1	2	2	3
C	Nodules/ulcers	0	0	3	2
	Onychogryphosis	0	0	1	3
	Dry exfoliative dermatitis/alopecia	0	0	0	3
O	Blepharitis	0	0	0	1
	Keratoconjunctivitis	0	0	0	0
	Uveitis	0	0	0	0
CP	Anemia	3	5	9	27
	Thrombocytopenia^c^	1	6	19	33
	Total protein increase	0	3	6	18
	Albumin/globulin ratio inversion	0	4	12	26
	Creatinine increase	0	0	2	4
	Urea increase	0	1	5	11

Note that more than one sign could be present in the same dog.

a Including 3 dogs dead from CanL between M12 and M18;

b Including 2 dogs dead from CanL between M18 and M22;

c Severe thrombocytopenia developed in 12 dogs from study A was most probably caused by *Ehrlichia* co-infection.

### Study D

**Figure 3:**
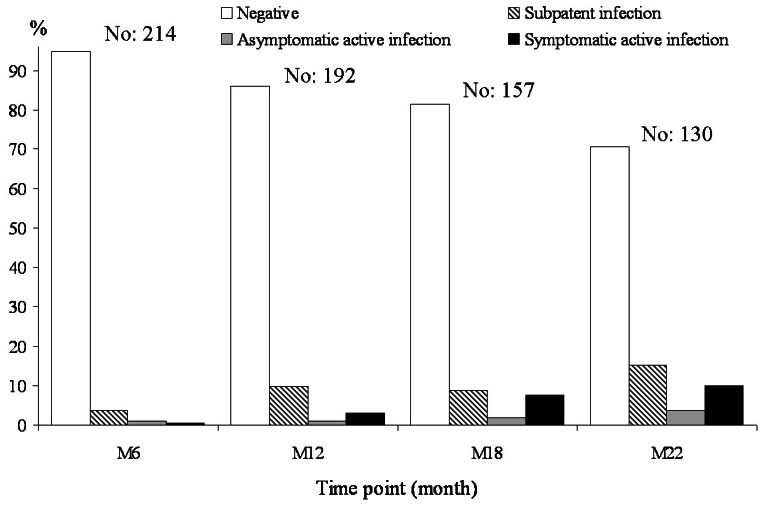
Prospective study D. Incidence of *Leishmania* infection stages detected in 214 owned dogs of different breeds at four time points from natural exposure.

A total of 130/214 dogs completed the study by attending regular follow-up examinations; 86 dogs were lost during the follow-up either because of poor owner's compliance to attend examinations, or due to deaths unrelated to *Leishmania* infection. At our best knowledge, none of the lost dogs have developed or died from symptomatic CanL. Infection stages recorded at each time point are shown in Fig. 3. At M22 92 dogs were still negative, 20 were in a sub-patent infection stage, 5 showed asymptomatic active infection and 13 symptomatic active infection, for a cumulative clinical disease incidence of 10.0% in about 2 years from initial exposure. Subpatent infections detected at each assessment showed a variety of progression patterns similar to A–C studies (data not shown). Clinically susceptible dogs belonged to 6 out of 29 breeds included initially in the study: 5 were mongrel dogs out of 70, 1/27 English Setters, 1/5 Beagles, 1/5 Bretons, 2/3 Bull dogs and 3/4 Dobermanns.

For the 13 dogs that developed a symptomatic infection, individual clinical and clinicopathological signs were recorded at the time of their appearance and categorized as done previously (Table 2). In this study the frequency and variety of clinical signs also increased during time. Only one dog exhibited a pathological sign at M6 (thrombocytopenia), whereas several signs were recorded from M12. Similarly to what observed in A–C studies, the most frequent sign was the enlargement of lymph nodes that involved 85% of sick dogs by the end of the study. Analogously, cutaneous signs were mostly late and much less frequent, and ocular ones very rare and late being recorded in one dog only at M22. Anemia and thrombocytopenia were the most common laboratory alterations and found in about half of the dogs. All other alterations appeared from M18 and were less frequent.

**Table 2:** Incidence of clinical and clinicopathological signs, prospective study D.

Sign category	Individual sign	No. of dogs showing signs and no. of signs recorded at each time point (M)			
		No = 1 (M6)	No = 6 (M12)	No = 12 (M18)	No = 13 (M22)
S	Weight loss	0	2	2	2
	Muscular atrophy	0	0	0	0
	Lethargy	0	0	0	0
	Pale mucous membranes	0	0	1	0
RE	Lymph node enlargement	0	6	8	11
	Splenomegaly	0	0	0	0
C	Nodules/ulcers	0	0	1	4
	Onychogryphosis	0	0	1	1
	Dry exfoliative dermatitis/alopecia	0	2	2	2
O	Blepharitis	0	0	0	0
	Keratoconjunctivitis	0	0	0	1
	Uveitis	0	0	0	0
CP	Anemia	0	0	2	6
	Thrombocytopenia	1	1	4	6
	Total protein increase	0	0	4	4
	Albumin/globulin ratio inversion	0	0	3	3
	Creatinine increase	0	0	1	4
	Urea increase	0	0	4	4

Note that more than one sign could be present in the same dog.

### Treated *versus* control dogs in studies A, C and D resulted in vaccine failure

In order to justify the unequivocal inclusion of dogs treated with non-effective vaccines in our clinical analysis, we needed to verify if the exposure to *Leishmania* antigens in the context of an adjuvant may have altered the appearance and progression of the variety of clinical signs in vaccinated as compared to untreated dogs. In Beagles of combined A and C studies, 7 vaccinated and 3 controls at M12, 9 vaccinated and 5 controls at M18, and 19 vaccinated and 12 controls at M22 developed a number and type of clinical and clinicopathological signs at similar frequency distribution at each time point (p = 0.68) (data not shown). Quite similar results (p = 0.66, data not shown) were seen for the dogs of study D, which involved 3 vaccinated and 4 controls at M12, 4 vaccinated and 8 controls at M18, and 4 vaccinated and 9 controls at M22.

### Comparison of infection and clinical parameters between combined A, B and C studies, and study D

The most striking differences between the two sets of studies were in dog breed composition (Beagles representing 100% and 2.3% of dogs in the two study sets) and the incidence of *Leishmania* transmission to which the naïve dogs have been exposed. In fact, the M22 point-prevalence of all infection stages, including deaths from CanL, was 64/107 (59.8%) in combined A–C studies, while it was just half of that in study D (38/130, 29.2%) (p<0.001). Because the progression from subpatent to active infection stages (a major indication of susceptibility) could theoretically be influenced also by exposure to elevated forces of *Leishmania* infection as a result of the host's immunological impairment due to repeated antigenic stimulations, we have compared the relative proportion of active infections recorded at each time point between the two study sets. As shown in Table 3, these proportions were very similar in both groups of dogs from M6 to M18, with a tendency of being lower in dogs of study D at M22 although not statistically relevant. We then compared the patterns of clinical and clinicopathological signs developed at M22 in the two groups of dogs like they appear in Tables 1 and 2. As shown in Table 4, the frequency distribution of sign categories did not differ significantly between the two study sets. Similar results were obtained when frequencies of each individual sign were compared, except for the rates of thrombocytopenia and albumin/globulin ratio inversion that were found significantly higher in dogs of combined A–C studies (p = 0.009 and p = 0.011, respectively). At least for severe thrombocytopenia, it should be pointed out that 12 dogs from study A suffered from acute or chronic *E. canis* infection, a major cause of this pathological condition.

**Table 3:** Proportion of dogs with active infections over all infected dogs.

Study	M6	M12	M18	MT22
Combined A, B & C	6/16 (37.5%)	13/42 (31.0%)	24/42 (57.1%)^b^	43/64 (67.2%)^c^
D	3/11 (27.3%)	8/27 (29.6%)	15/29 (51.7%)	18/38 (47.4%)
p value^a^	0.45	0.88	0.83	0.08

a At the Fisher's exact test or Chi-square test where appropriate.

b Including 3 dogs dead from CanL between M12 and M18.

c Including all dogs (5) dead from CanL through M22.

**Table 4:** Frequency of clinical and clinicopathological sign categories recorded at M22.

Study	No. of dogs	S	RE	C	O	CP
Combined A, B & C	43	0.37	0.60	0.21	0.02	3.13
D	13	0.15	0.85	0.54	0.08	2.08
p value^a^	0.24					

Note that the frequency value can be >1 because more than one sign could be present in the same dog. **S**: systemic signs; **RE**: reticulo-endothelial signs; **C**: cutaneous signs; **O**: ocular signs; **CP**: clinicopathological signs.

a At the Mann-Whitney test (paired test).

**Figure 4:**
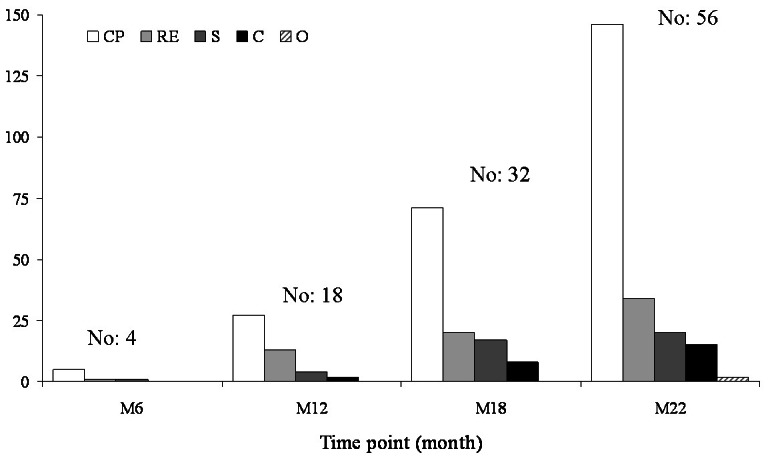
All prospective studies. Number of clinical signs belonging to 5 categories as recorded at each time point in 56 dogs that developed canine leishmaniasis disease (data from Tables 1 and 2). Note that more than one sign could be present in the same dog. **CP**: clinicopathological signs; **RE**: reticulo-endothelial signs; **S**: systemic signs; **C**: cutaneous signs; **O**: ocular signs.

Altogether our findings indicate that the canine populations from the 2 study sets behaved in similar ways in the response to natural *Leishmania* infections. We may conclude that once susceptible young dogs are infected by *L. infantum* they have the same proportionate risk of progressing to advanced stages whatever the breed and the infection incidence, presenting a common pattern of clinical signs during 2 years post infection (Fig. 4).

## Discussion

We have confirmed in a larger number of dogs a model of progression of natural CanL infections described previously in a limited sample of Beagles [10]. Our model is based on a standard set of diagnostic markers for infection which may be considered somewhat limited as compared with those investigated in dogs that have undergone necropsy; for example, no skin samples were examined, and culture and PCR were performed from two different single tissues (lymph node and bone marrow, respectively). This approach was mainly dictated by the frequent repetition of samplings - several other assessments were indeed performed which were not included in our analysis because not concordant in periodicity among different studies - and by lower invasiveness of procedures as required by the ethical committees that approved the studies. Once we have evaluated that nested-PCR could be better applied to a homogeneous material like the bone marrow aspirate, considered more appropriate for legitimate comparisons between vaccine/control groups in regards to initial infections, the low amount of sample material obtained from one bone (about 0.4 mL) [9] could not be employed also for culture purposes. On the other hand, *Leishmania* culture yields from lymph node aspirates are thought to be similar to those from bone marrow aspirates, as reported by several authors (see [3] for a review).

According to our model, from a prospective point of view subpatent infections may or may not progress to a stage of active infection and in some cases they may convert to negative. On the contrary, once established, active infections appear to not regress towards negative or subpatent conditions. Furthermore, the asymptomatic condition of actively infected dogs is only temporary and the progression towards overt disease seems to be the rule. The present study fully supports this pattern in different epidemiological situations and among different dog breeds. We have shown that any susceptible young dogs exposed to different transmission incidences have similar chance to develop an active infection and become symptomatic. We use the term “susceptible” not necessarily associated to a specific dog breed but rather to a genetic make-up, although the D study identified a rank of active infection incidences differently distributed among dogs initially enrolled and belonging to 6 breeds, from 1/27 English Setters (3.7%) to 3/4 Dobermanns (75.0%). Considering the “disperse” nature of study D, a deeper investigation on CanL susceptibility associated to breed would require thorough analysis of several risk factors besides breed itself (such as the local incidence of transmission in 12 sites, occupational and environmental factors, appropriate denominators for each breed at each time point and site, etc.) which is far from the scope of this paper.

This study also demonstrates that any susceptible dogs naturally infected by *L. infantum* exhibit a common pattern of progression of clinical signs during 2 years post infection. Sick dogs showed the first clinical and clinicopathological signs after 6 months from exposure. During follow up, the progression of the disease was characterized by a deterioration of the dog's condition with the increase in variety and severity of signs. The most representative signs were in the clinicopathological, reticulo-endothelial and systemic categories. The almost regular finding of lymph-node enlargement confirms previous cross-sectional studies where generalized lymphadenomegaly was recorded as the most frequent sign of CanL [2], [17]. On the other hand, cutaneous signs were rare and ocular ones almost negligible during the first 22 months. It is well known that major and severe clinical signs of CanL are attributable to the deposition of immune complexes that may cause glomerulonephritis, vasculitis, polyarthritis, uveitis and meningitis [18]–[21]. The rarity of cutaneous and ocular signs, which are also caused by these pathological alterations, suggests that a chronic process would normally require more than 2 years post infection before being clinically manifest. The time course of serum protein and biochemical alterations further confirmed this hypothesis, since the markers for renal deterioration in our dog cohorts became apparent only 4–6 months after first recordings of hypergammaglobulinemia. On the contrary, the earliest and most frequent clinicopathological sign was thrombocytopenia, for which different pathogenic mechanisms have been demonstrated [22], [23]. Non-regenerative anemia showed similar frequency and longitudinal trend, which indicates a major involvement of bone marrow, a tissue found heavily parasitized in dogs developing disease. Due to the detection of anti-*Ehrlichia* antibodies in some dogs included in study A, we cannot exclude a combined pathological effect by the two agents as described in a previous study [16].

Our study may provide clues for medical as well as epidemiological applied aspects, such as the clinical monitoring of dogs found temporarily or permanently at *Leishmania* infection risk, the therapeutic management of infected dogs, or the clinical efficacy evaluation of preventive measures such as anti-*Leishmania* vaccines or topical repellents/insecticides against phlebotomine sand flies. Furthermore, several studies on dog's infectiousness to competent vectors performed through xenodiagnosis have so far included comparisons between dog groups broadly defined as “asymptomatic” and “symptomatic”. However, neither accurate staging of the infection (e.g. discrimination between subpatent or active infection in dogs without clinical signs), nor the inclusion of clinicopathological alterations for the “symptomatic” definition, nor the appraisal of clinical progression stages (e.g. early, without skin alterations; or late/chronic, including such alterations) were usually performed [4]–[6], [24], [25]. Results from the above studies have been often controversial as regards the infectiousness capacity of “asymptomatic” groups, probably as a result of the mix-up of various infection and clinical conditions differently represented in the study groups. Hence, our study can provide clues for a better standardization of comparative xenodiagnosis studies, which are of paramount importance for targeting infectious dogs in the control of zoonotic visceral leishmaniasis [26].

## References

[1] Gramiccia M, Gradoni L (2005) The current status of zoonotic leishmaniases and approaches to disease control. Int J Parasitol 35: 1169–1180. 16162348 10.1016/j.ijpara.2005.07.001

[2] Ciaramella P, Oliva G, De Luna R, Gradoni L, Ambrosio R, et al (1997) A retrospective clinical study of canine leishmaniasis in 150 dogs naturally infected by *Leishmania infantum* . Vet Rec 141: 539–543. 9413121 10.1136/vr.141.21.539

[3] Alvar J, Cañavate C, Molina R, Moreno J, Nieto J (2004) Canine leishmaniasis. Adv Parasitol 57: 1–88. 15504537 10.1016/S0065-308X(04)57001-X

[4] Courtenay O, Quinnell RJ, Garcez LM, Shaw JJ, Dye C (2002) Infectiousness in a cohort of Brazilian dogs: why culling fails to control visceral leishmaniasis in areas of high transmission. J Infect Dis 186: 1314–1320. 12402201 10.1086/344312

[5] Michalsky EM, Rocha MF, Da Rocha Lima AC, França-Silva JC, Pires MQ, et al (2007) Infectivity of seropositive dogs, showing different clinical forms of leishmaniasis, to *Lutzomyia longipalpis* phlebotomine sand flies. Vet Parasitol 147: 67–76. 17449184 10.1016/j.vetpar.2007.03.004

[6] Molina R, Amela C, Nieto J, San-Andrés M, González F, et al (1994) Infectivity of dogs naturally infected with *Leishmania infantum* to colonized *Phlebotomus perniciosus* . Trans R Soc Trop Med Hyg 88: 491–493. 7570854 10.1016/0035-9203(94)90446-4

[7] Solano-Gallego L, Koutinas A, Miró G, Cardoso L, Pennisi MG, et al (2009) Directions for the diagnosis, clinical staging, treatment and prevention of canine leishmaniosis. Vet Parasitol 165: 1–18. 19559536 10.1016/j.vetpar.2009.05.022

[8] Oliva G, Roura X, Crotti A, Maroli M, Castagnaro M, et al (2010) Guidelines for treatment of leishmaniasis in dogs. J Am Vet Med Assoc 236: 1192–1198. 20513197 10.2460/javma.236.11.1192

[9] Gradoni L, Foglia Manzillo V, Pagano A, Piantedosi D, De Luna R, et al (2005) Failure of a multi-subunit recombinant leishmanial vaccine (MML) to protect dogs from *Leishmania infantum* infection and to prevent disease progression in infected animals. Vaccine 23: 5245–5251. 16054272 10.1016/j.vaccine.2005.07.001

[10] Oliva G, Scalone A, Foglia Manzillo V, Gramiccia M, Pagano A, et al (2006) Incidence and time course of *Leishmania infantum* infections examined by parasitological, serologic, and nested-PCR techniques in a cohort of naïve dogs exposed to three consecutive transmission seasons. J Clin Microbiol 44: 1318–1322. 16597857 10.1128/JCM.44.4.1318-1322.2006 PMC1448675

[11] Oliva G, Nieto J, Foglia Manzillo V, Cappiello S, Fiorentino E, et al. (2011) Evidence for protection against active infection and disease progression in naïve dogs vaccinated with LiESP/QA-21 (CaniLeish®) exposed to two consecutive *Leishmania infantum* transmission seasons. Proceedings of the WSAVA/FECAVA/BSAVA World Congress, Birmingham (UK), 11–15 April 2012, p. 529–530.

[12] Maroli M, Mizzoni V, Siragusa C, D'Orazi A, Gradoni L (2001) Evidence for an impact on the incidence of canine leishmaniasis by the mass use of deltamethrin-impregnated dog collars in southern Italy. Med Vet Entomol 15: 358–263. 11776454 10.1046/j.0269-283x.2001.00321.x

[13] Maroli M, Gradoni L, Oliva G, Castagnaro M, Crotti A, et al (2010) Guidelines for prevention of leishmaniasis in dogs. J Am Vet Med Assoc 236: 1200–1206. 20513198 10.2460/javma.236.11.1200

[14] Gradoni L, Gramiccia M (2008) Leishmaniosis. In: OIE Manual of Diagnostic tests and vaccines for terrestrial animals (mammals, birds and bees). 6th edition. Paris: Office International des Epizooties. p. 240–250.

[15] Van Eys GJJM, Schoone GJ, Kroon MNC, Ebeling SB (1992) Sequence analysis of small subunit ribosomal RNA genes and its use for detection and identification of *Leishmania* parasites. Mol Biochem Parasitol 51: 133–142. 1565128 10.1016/0166-6851(92)90208-2

[16] Mekuzas Y, Gradoni L, Oliva G, Foglia Manzillo V, Baneth G (2009) *Ehrlichia canis* and *Leishmania infantum* co-infection: a 3-year longitudinal study in naturally exposed dogs. Clin Microbiol Infect 15 (Suppl. 2)30–31. 19416288 10.1111/j.1469-0691.2008.02150.x

[17] Koutinas AF, Polizopoulou ZS, Saridomichelakis MN, Argyriadis D, Fytianou A, et al (1999) Clinical considerations on canine visceral leishmaniasis in Greece: a retrospective study of 158 cases (1989–1996). J Am Anim Hosp Assoc 35: 376–383. 10493412 10.5326/15473317-35-5-376

[18] García-Alonso M, Blanco A, Reina D, Serrano FJ, Alonso C, et al (1996) Immunopathology of the uveitis in canine leishmaniasis. Parasite Immunol 18: 617–623. 9226700 10.1046/j.1365-3024.1996.d01-39.x

[19] Nieto CG, Navarrete I, Habela MA, Serrano F, Redondo E (1992) Pathological changes in kidneys of dogs with natural *Leishmania* infection. Vet Parasitol 45: 33–47. 1485420 10.1016/0304-4017(92)90025-5

[20] Paltrinieri S, Solano-Gallego L, Fondati A, Lubas G, Gradoni L, et al (2010) Guidelines for diagnosis and clinical classification of leishmaniasis in dogs. J Am Vet Med Assoc 236: 1184–1191. 20513195 10.2460/javma.236.11.1184

[21] Poli A, Abramo F, Mancianti F, Nigro M, Pieri S, et al (1991) Renal involvement in canine leishmaniasis. A light-microscopic, immunohistochemical and electron-microscopic study. Nephron 57: 444–52. 2046828 10.1159/000186348

[22] Cortese L, Sica M, Piantedosi D, Ruggiero G, Pero ME, et al (2009) Secondary immune-mediated thrombocytopenia in dogs naturally infected by *Leishmania infantum* . Vet Rec 164: 778–782. 19542552 10.1136/vr.164.25.778

[23] Foglia Manzillo V, Restucci B, Pagano A, Gradoni L, Oliva G (2006) Pathological changes in the bone marrow of dogs with leishmaniosis. Vet Rec 158: 690–694. 16714433 10.1136/vr.158.20.690

[24] Soares MR, de Mendonça IL, do Bonfim JM, Rodrigues JA, Wernek GL, et al (2011) Canine visceral leishmaniasis in Teresina, Brazil: relationship between clinical features and infectivity for sand flies. Acta Trop 117: 6–9. 20816657 10.1016/j.actatropica.2010.08.015

[25] Travi BL, Tabares CJ, Cadena H, Ferro C, Osório Y (2001) Canine visceral leishmaniasis in Colombia: relationship between clinical and parasitological status and infectivity for sand flies. Am J Trop Med Hyg 64: 119–124. 11442205 10.4269/ajtmh.2001.64.119

[26] World Health Organization (2010) Control of the leishmaniases: Report of a meeting of the WHO Expert Committee on the Control of Leishmaniases, Geneva, 22–26 March 2010, Tech Rep Ser 949. Geneva: WHO press.

